# Urgent surgery after emergency presentation for colorectal cancer has no impact on overall and disease-free survival: a propensity score analysis

**DOI:** 10.1186/s12885-016-2239-8

**Published:** 2016-03-11

**Authors:** Benjamin Weixler, Rene Warschkow, Michaela Ramser, Raoul Droeser, Urs von Holzen, Daniel Oertli, Christoph Kettelhack

**Affiliations:** Department of Surgery, University Hospital Basel, Spitalstrasse 21, 4031 Basel, Switzerland; Department of Surgery, Kantonsspital St. Gallen, 9007 St. Gallen, Switzerland; Institute of Medical Biometry and Informatics, University of Heidelberg, Heidelberg, Germany; Goshen Center for Cancer Care, Goshen, IN 46507 USA

**Keywords:** Colorectal cancer, Emergency surgery, Oncological outcome, Overal survival, Disease-free survival

## Abstract

**Background:**

It remains a matter of debate whether colorectal cancer resection in an emergency setting negatively impacts on survival. Our objective was therefore to assess the impact of urgent versus elective operation on overall and disease-free survival in patients undergoing resection for colorectal cancer by using propensity score adjusted analysis.

**Methods:**

In a single-center study patients operated for colorectal cancer between 1989 and 2013 were identified from a prospectively maintained database. Median follow-up was 44 months. Patients with neoadjuvant treatment were excluded. The impact of urgent operation on overall and disease-free survival was assessed using both Cox regression and propensity score analyses.

**Results:**

Of 747 patients with colorectal cancer, 84 (11 %) had urgent and 663 elective cancer resection. The propensity score revealed strongly biased patient characteristics (0.22 ± 0.16 vs. 0.10 ± 0.09; *P* < 0.001). In unadjusted analysis urgent operation was associated with a 35 % increased risk of overall mortality (hazard ratio(HR) of death = 1.35, 95 % confidence interval(CI):1.02–1.78, *P* = 0.045). In risk-adjusted Cox regression analysis urgent operation was not associated with poor overall (HR = 1.08, 95 %CI:0.79–1.48; *P* = 0.629) or disease-free survival (HR = 1.02, 95 %CI:0.76–1.38; *P* = 0.877). Similarly in propensity score analysis urgent operation did not influence overall (HR = 0.98, 95 % CI:0.74–1.29), *P* = 0.872) and disease-free survival (HR = 0.89, 95 %CI:0.68 to 1.16, *P* = 0.387).

**Conclusions:**

This study provides evidence that worse oncologic outcomes after urgent operation for colorectal cancer are caused by clinical circumstances and not due to the urgent operation itself. Urgent operation is not a risk factor for colorectal cancer resection.

## Background

Colorectal cancer remains one of the most prevalent malignancies worldwide and a leading cause of cancer related death. Surgical resection including systematic lymphadenenctomy is the treatment of choice. Unfortunately, only half of these curatively operated patients will survive beyond five years. Up to 30 % of colorectal cancer patients are first diagnosed during emergency department presentation due to symptomatic disease [1–3]. Previous studies demonstrated that mortality rates are as much as four times higher for the immediate postoperative period in patients undergoing urgent operation. Results concerning long time survival are more conflicting [1–10]. However, many studies report on rather small sample sizes, state only immediate postoperative mortality rates or do not compare their results with a comparative group of electively operated patients [4, 9, 11–14]. Retrospective study design as well as a small percentage of patients presenting as an emergency make potential bias very likely. Randomisation could eliminate such bias but is not applicable for these symptomatic patients. Propensity score matching accounts for such bias in nonrandomized studies by eliminating different distribution of observed variables between two groups.

The objective of this study was to assess the impact of urgent surgery on oncologic outcomes in a large homogenic cohort of colorectal cancer patients. Both Cox proportional hazard regression analyses as well as propensity-scoring methods were used.

## Methods

Data for the present retrospective study were extracted from the prospectively maintained cancer registry database at our institution, a tertiary care center in Switzerland. Overall, 830 patients undergoing colorectal cancer resection between 1989 and 2013 were identified. Eighty patients with neoadjuvant therapy were excluded as were three patients who were lost to follow-up. 747 patients remained for further analyses. Two groups were compared, 84 patients with urgent operation and 663 patients who did undergo elective cancer resection. The study was approved by the local ethical committee (Ethikkommission Nordwest- und Zentralschweiz). Follow-up data were collected from the treating general practitioner of the respective patients. Approval of data collection was obtained prior to surgery in years 1989–2005. For patients operated between 2006 and 2013 consent was obtained via letters of enquiry that were sent to these patients.

### Data collection and definitions

Data on patients’ demographics, mode of presentation, operative details, postoperative mortality and histological results were collected from the patients case notes. All operations were performed or supervised by experienced colorectal surgeons. Definition of urgent surgery was used according to the NCEPOD classification of intervention (e.g., immediate (within minutes), urgent (<hours), expedited (<days) and elective (planned)) [15]. For the purpose of this study, patients undergoing immediate or urgent operations were grouped as urgent surgery. However, no patients underwent immediate surgery within minutes after emergency department presentation.

According to the postoperative staging adjuvant chemotherapy was administered routinely in patients with node positive disease. Follow-up and recurrence data could be almost entirely collected from our clinical records, or the bureau of vital statistics and the treating physician, respectively.

### Statistical analyses

Statistical analyses were performed using the R statistical software (www.r-project.org). A two-sided *p*-value < 0.05 was considered statistically significant. Continuous data are expressed as means ± standard deviation. For comparing proportions, Chi-Square statistics and for comparing continuous variable, t-tests and Mann–Whitney U-tests were used as appropriate. Missing data were imputed using the random survival forest method [16].

First, the bias concerning elective versus urgent operation was assessed regarding age, gender, tumor localisation, tumor stage, and adjuvant therapy. The same set of covariates, including elective versus urgent operation were then assessed as putative prognostic factors for overall and disease-free survival in unadjusted and risk-adjusted Cox regressions, including a backward variable selection procedure from the full Cox regression model based on the Akaike’s information criterion. Moreover, a propensity score analysis as a superior and more refined statistical method of adjusting for potential baseline confounding variables was performed [17–20]. We used the “Matching” R package to perform a bipartite weighting propensity score analysis [21, 22]. The baseline risk profiles of the matched patients were compared to assure that no major differences in baseline patients characteristics persisted. The prognostic value of elective versus urgent operation for overall and disease-free survival was finally assessed in a stratified Cox regression analysis applying the subclasses and the weights obtained by the propensity score analysis.

## Results

### Patient characteristics and bias in urgent versus elective operation

747 patients with a median follow-up time of 44 months (range 0–247 months, mean 63.5 months) were eligible for the present analysis. 84 patients underwent urgent operation and 663 patients had elective cancer resection as defined above. The 30 day postoperative mortality rate was 5.2 % (35 of 663 patients) following curative resection and 8.3 % (7 of 84 patients) after urgent colorectal cancer resection. In more than 90 % of patients complete resection of the tumor could be achieved and about half of the patients presented with node positive disease (49.4 %, *n* = 369). Table 1 summarizes the characteristics of patients with urgent and elective cancer resection. In univariate analysis tumor localisation, perforation, resection status and number of extracted lymph nodes significantly differed between patients with urgent and elective operation (Table 1). After multivariable adjustment, number of extracted lymph nodes was associated with urgent surgery and perforation was an independent statistically significant predictor for urgent operation (Table 1). Other differences in the patient characteristics failed to reach the significance level.

**Table 1:** Patient characteristics and bias for urgent versus elective operation

		Patient characteristics in univariate analysis				Bias in multivariable logistic regression		Patient characteristics after propensity score matching		
		Total *N* = 747	Urgent *N* = 84	Elective *N* = 663	*p*	OR (95 % CI)	*p* ^c^	Urgent *N* = 83	Elective *N* = 621	*p*
Age	years	71.4 ± 12.1	72.0 ± 11.2	71.3 ± 12.2	0.884^a^	1.01 (0.99–1.03)	0.490	72.1 ± 11.3	72.5 ± 11.7	0.760^a^
Sex	m	421 (56.4 %)	43 (51.2 %)	378(57.0 %)	0.311^b^	Reference	0.517	42 (50.6 %)	333.5 (53.7 %)	0.594^b^
	w	326 (43.6 %)	41 (48.8 %)	285 (43.0 %)		1.19 (0.71–1.99)		41 (49.4 %)	287.5 (46.3 %)	
Tumor localisation	Cecum	132 (17.7 %)	12 (14.3 %)	120 (18.1 %)	0.019^b^	Reference	0.092	12 (14.5 %)	95.4 (15.4 %)	0.927^b^
	Ascending colon	130 (17.4 %)	12 (14.3 %)	118 (17.8 %)		1.25 (0.50–3.19)		12 (14.5 %)	82.4 (13.3 %)	
	Transverse colon	40 (5.4 %)	7 (8.3 %)	33 (5.0 %)		2.50 (0.80–7.43)		7 (8.4 %)	55.7 (9.0 %)	
	Descending colon	81 (10.8 %)	12 (14.3 %)	69 (10.4 %)		2.29 (0.88–6.01)		12 (14.5 %)	80.9 (13.0 %)	
	Sigmoid colon	201 (26.9 %)	32 (38.1 %)	169 (25.5 %)		1.95 (0.90–4.44)		31 (37.3 %)	258.1 (41.6 %)	
	Rectum	163 (21.8 %)	9 (10.7 %)	154 (23.2 %)		0.78 (0.28–2.12)		9 (10.8 %)	48.7 (7.8 %)	
Perforation	No	674 (90.2 %)	56 (66.7 %)	618 (93.2 %)	<0.001^b^	Reference	<0.001	56 (67.5 %)	465.5 (75.0 %)	0.144^b^
	Yes	73 (9.8 %)	28 (33.3 %)	45 (6.8 %)		7.17 (3.93–13.09)		27 (32.5 %)	155.5 (25.0 %)	
Protective colostomy	No	657 (88.0 %)	71 (84.5 %)	586 (88.4 %)	0.306^b^	Reference	0.129	70 (84.3 %)	512.7 (82.6 %)	0.687^b^
	Yes	90 (12.0 %)	13 (15.5 %)	77 (11.6 %)		1.82 (0.83–3.80)		13 (15.7 %)	108.3 (17.4 %)	
Resection status	R0	718 (96.1 %)	76 (90.5 %)	642 (96.8 %)	0.014^c^	Reference	0.114	76 (91.6 %)	571.8 (92.1 %)	0.870^b^
	R1/2	29 (3.9 %)	8 (9.5 %)	21 (3.2 %)		2.32 (0.81–6.09)		7 (8.4 %)	49.2 (7.9 %)	
UICC Stage	I	166 (22.2 %)	13 (15.5 %)	153 (23.1 %)	0.332^b^	Reference	0.599	13 (15.7 %)	69.9 (11.3 %)	0.698^b^
	II	212 (28.4 %)	23 (27.4 %)	189 (28.5 %)		1.41 (0.63–3.29)		23 (27.7 %)	172.4 (27.8 %)	
	III	220 (29.5 %)	27 (32.1 %)	193 (29.1 %)		1.30 (0.55–3.15)		26 (31.3 %)	209.8 (33.8 %)	
	IV	149 (19.9 %)	21 (25.0 %)	128 (19.3 %)		1.83 (0.72–4.71)		21 (25.3 %)	168.9 (27.2 %)	
Tumor diameter	mm	45.8 ± 21.6	45.7 ± 20.6	45.8 ± 21.7	0.831^a^	0.99 (0.98–1.00)	0.107	45.6 ± 20.7	43.7 ± 19.3	0.421^a^
Lymph node yield	<12	166 (22.2 %)	10 (11.9 %)	156 (23.5 %)	0.016^b^	Reference	0.040	10 (12.0 %)	46.1 (7.4 %)	0.143^b^
	12+	581 (77.8 %)	74 (88.1 %)	507 (76.5 %)		2.08 (1.03–4.58)		73 (88.0 %)	574.9 (92.6 %)	
Tumor grading	G1	23 (3.1 %)	3 (3.6 %)	20 (3.0 %)	0.180^b^	Reference	0.196	3 (3.6 %)	29.2 (4.7 %)	0.823^b^
	G2	540 (72.3 %)	53 (63.1 %)	487 (73.5 %)		0.45 (0.14–2.03)		52 (62.7 %)	355.9 (57.3 %)	
	G3	148 (19.8 %)	21 (25.0 %)	127 (19.2 %)		0.62 (0.17–2.99)		21 (25.3 %)	175.1 (28.2 %)	
	GX	36 (4.8 %)	7 (8.3 %)	29 (4.4 %)		1.21 (0.26–6.73)		7 (8.4 %)	60.7 (9.8 %)	
Adjuvant Chemotherapy	No	505 (67.6 %)	51 (60.7 %)	454 (68.5 %)	0.152^b^	Reference	0.785	51 (61.4 %)	382.8 (61.6 %)	0.973^b^
	Yes	242 (32.4 %)	33 (39.3 %)	209 (31.5 %)		1.09 (0.59–2.01)		32 (38.6 %)	238.2 (38.4 %)	

n (%); mean ± standard deviation

Number of patients after elective operation with decimals because of weigthing in the propensity score matching analysis

^a^ Mann–Whitney *U*-test; ^b^ Chi-Square statistic; ^c^ Likelihood ratio test

### Urgent operation as a prognostic factor for overall survival

**Fig. 1:**
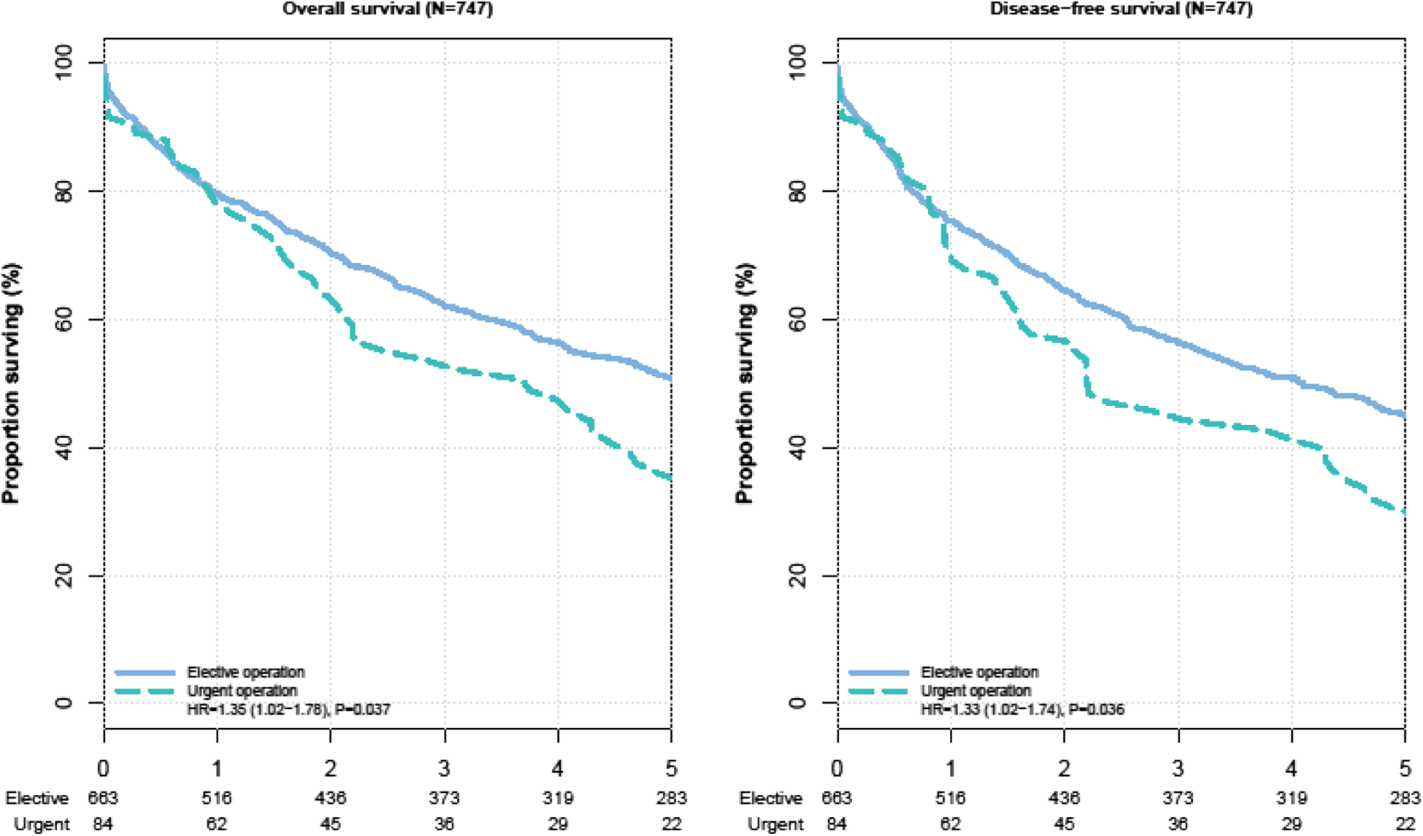
Kaplan–Meier curve for overall and disease-free survival in unadjusted analysis. The number of colorectal cancer patients at risk are given below each plot. Survival curves are provided with 95 % confidence intervals

An unadjusted Cox proportional hazards regression analysis revealed urgent operation as a statistically significant prognostic factor with an approximately 35 % increased risk of overall mortality (HR of death = 1.35, 95 % CI: 1.02 to 1.78, *P* = 0.045) and an approximately 33 % increased risk of disease recurrence (HR of event = 1.33, 95 % CI: 1.02 to 1.74, *P* = 0.043) (Table 2). The five-year overall survival for patients with urgent operation was 35.9 % (95 % CI: 26.1 to 49.4 %) compared to 50.8 % (95 % CI: 47.0 to 54.9 %) in patients with elective operation (Fig. 1, left panel). The five-year disease-free survival for patients with urgent operation was 30.6 % (95 % CI: 21.6 to 43.3 %) compared to 45.0 % (95 % CI: 41.2 to 49.1 %) in patients undergoing elective operation (Fig. 1, right panel). When adjusting for potential confounding factors in risk-adjusted Cox regression analyses, urgent operation did not influence overall survival (HR of death = 1.08, 95 % CI: 0.79 to 1.48; *P* = 0.629) or disease-free survival (HR of event = 1.02, 95 % CI: 0.76 to 1.38; *P* = 0.877). Elective versus urgent operation was excluded from the full Cox regression models based on the change in the Akaike’s information criterion as these two variables did not show relevant predictive value for OS and DFS(Table 2).

**Table 2:** Prognostic factors for overall and disease-free survival after colorectal cancer resection

Prognostic factors		Overall survival						Disease free survival					
		Unadjusted^a^		Full model^b^		Backwards variable selection^c^		Unadjusted^a^		Full model^b^		Backwards variable selection^c^	
		HR (95 % CI)	*p* *	HR (95 % CI)	p*	HR (95 % CI)	p*	HR (95 % CI)	p*	HR (95 % CI)	*p* *	HR (95 % CI)	p*
Cancer resection	Elective	Reference	0.045	Reference	0.629	–	–	Reference	0.043	Reference	0.877	–	–
	Urgent	1.35 (1.02–1.78)		1.08 (0.79–1.48)		–	–	1.33 (1.02–1.74)		1.02 (0.76–1.38)		–	–
Age	years	1.04 (1.03–1.05)	<0.001	1.05 (1.04–1.06)	<0.001	1.05 (1.04–1.06)	<0.001	1.03 (1.03–1.04)	<0.001	1.04 (1.03–1.05)	<0.001	1.04 (1.03–1.05)	<0.001
Sex	m	Reference	0.973	Reference	0.850	–	–	Reference	0.478	Reference	0.381	–	–
	w	1.00 (0.84–1.20)		0.98 (0.81–1.19)		–	–	0.94 (0.79–1.12)		0.92 (0.76–1.11)		–	–
Tumor localisation	Cecum	Reference	0.004	Reference	0.002	Reference	0.003	Reference	0.018	Reference	0.026	Reference	0.031
	Asc. colon	0.72 (0.53–0.96)		0.60 (0.44–0.82)		0.62 (0.46–0.83)		0.76 (0.57–1.02)		0.67 (0.49–0.91)		0.68 (0.50–0.91)	
	Transv. colon	0.81 (0.53–1.23)		0.62 (0.40–0.97)		0.63 (0.41–0.97)		0.78 (0.51–1.18)		0.62 (0.40–0.95)		0.63 (0.41–0.96)	
	Desc. olon	0.87 (0.62–1.22)		0.90 (0.63–1.28)		0.90 (0.64–1.27)		0.94 (0.68–1.31)		0.99 (0.70–1.39)		1.03 (0.74–1.45)	
	Sigm. colon	0.73 (0.56–0.95)		0.66 (0.50–0.88)		0.68 (0.51–0.89)		0.80 (0.62–1.04)		0.79 (0.60–1.05)		0.83 (0.63–1.08)	
	Rectum	0.56 (0.42–0.74)		0.57 (0.42–0.79)		0.58 (0.43–0.78)		0.62 (0.47–0.81)		0.70 (0.51–0.95)		0.74 (0.56–0.99)	
Perforation	No	Reference	0.525	Reference	0.492	–	–	Reference	0.684	Reference	0.704	–	–
	Yes	1.10 (0.82–1.49)		1.13 (0.80–1.58)		–	–	1.06 (0.79–1.42)		1.07 (0.77–1.48)		–	–
Protec. colostomy	No	Reference	0.929	Reference	0.530	–	–	Reference	0.987	Reference	0.783	–	–
	Yes	1.01 (0.77–1.33)		1.10 (0.81–1.50)		–	–	1.00 (0.77–1.31)		1.04 (0.77–1.41)		–	–
Resection status	R0	Reference	<0.001	Reference	0.001	Reference	<0.001	Reference	<0.001	Reference	0.004	Reference	0.003
	R1/2	3.21 (2.16–4.75)		2.22 (1.45–3.38)		2.30 (1.52–3.49)		3.07 (2.08–4.55)		1.97 (1.29–3.02)		1.97 (1.30–3.00)	
UICC Stage	I	Reference	<0.001	Reference	<0.001	Reference	<0.001	Reference	<0.001	Reference	<0.001	Reference	<0.001
	II	1.28 (0.97–1.69)		1.21 (0.89–1.64)		1.25 (0.93–1.67)		1.41 (1.08–1.85)		1.39 (1.03–1.87)		1.37 (1.03–1.82)	
	III	1.67 (1.27–2.19)		1.67 (1.22–2.29)		1.74 (1.29–2.36)		1.80 (1.38–2.34)		1.79 (1.31–2.43)		1.75 (1.30–2.36)	
	IV	4.02 (3.02–5.35)		4.89 (3.45–6.94)		5.10 (3.64–7.14)		4.83 (3.65–6.40)		5.64 (3.99–7.97)		5.58 (4.00–7.79)	
Tumor diameter	mm	1.00 (1.00–1.01)	0.071	1.00 (1.00–1.01)	0.730	–	–	1.00 (1.00–1.01)	0.043	1.00 (1.00–1.01)	0.829	–	–
Lymph node yield	<12	Reference	0.662	Reference	0.006	Reference	0.007	Reference	0.662	Reference	0.173	–	–
	12+	0.95 (0.77–1.18)		0.72 (0.57–0.91)		0.73 (0.58–0.91)		1.05 (0.85–1.29)		0.85 (0.68–1.07)		–	–
Tumor grading	G1	Reference	0.012	Reference	0.367	–	–	Reference	0.001	Reference	0.009	Reference	0.007
	G2	1.09 (0.65–1.82)		1.23 (0.72–2.09)		–	–	1.07 (0.65–1.77)		1.21 (0.72–2.03)		1.20 (0.72–2.00)	
	G3	1.58 (0.92–2.71)		1.41 (0.80–2.47)		–	–	1.66 (0.98–2.81)		1.45 (0.84–2.50)		1.42 (0.83–2.45)	
	GX	1.33 (0.69–2.57)		1.59 (0.81–3.14)		–	–	1.57 (0.84–2.95)		2.44 (1.27–4.68)		2.49 (1.31–4.73)	
Adjuvant chemo.	No	Reference	0.679	Reference	0.042	Reference	0.027	Reference	0.501	Reference	0.142	Reference	0.142
	Yes	0.96 (0.79–1.17)		0.77 (0.60–0.99)		0.76 (0.60–0.97)		1.07 (0.88–1.29)		0.84 (0.66–1.06)		0.84 (0.66–1.06)	

*HR* Hazard ratios with 95 % confidence intervals (Wald type) and *p*-values of the likelihood ratio test

Prognostic factors for overall survival in:

*p values for likelihood ratio tes

^a^one Cox proportional hazards regression analyses for each factor

^b^Cox proportional hazards regression analyses for all factors

^c^Cox proportional hazards regression analyses for all factors after backwards variable selection

### Propensity score analysis

**Fig. 2:**
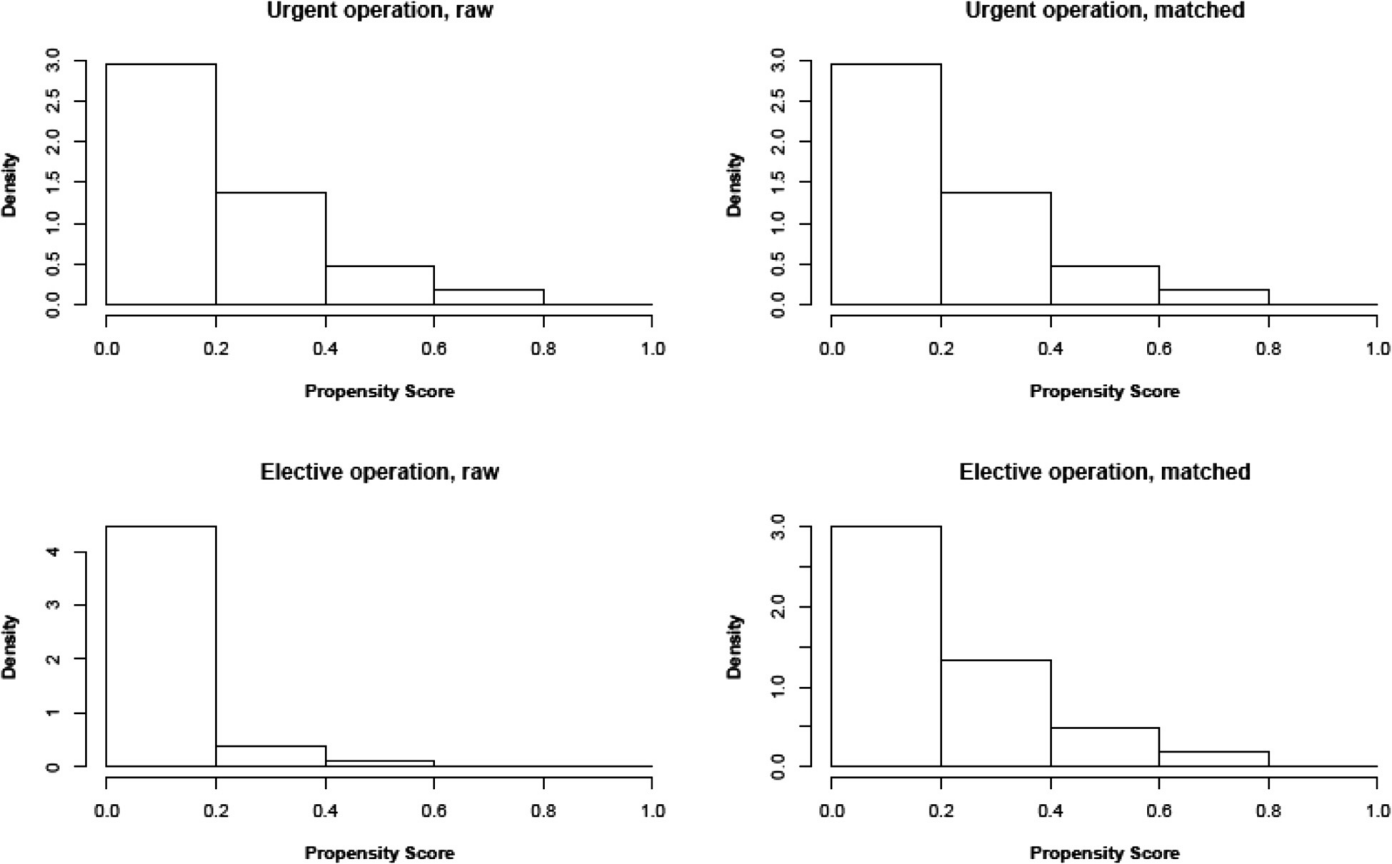
Distribution of propensity scores before and after propensity score analysis. The left upper and lower panels show the distribution of the propensity scores for patients with urgent and elective operation before the matching procedure. The right upper and lower panels demonstrate the distribution of the propensity scores after bipartite propensity score matching

**Fig. 3:**
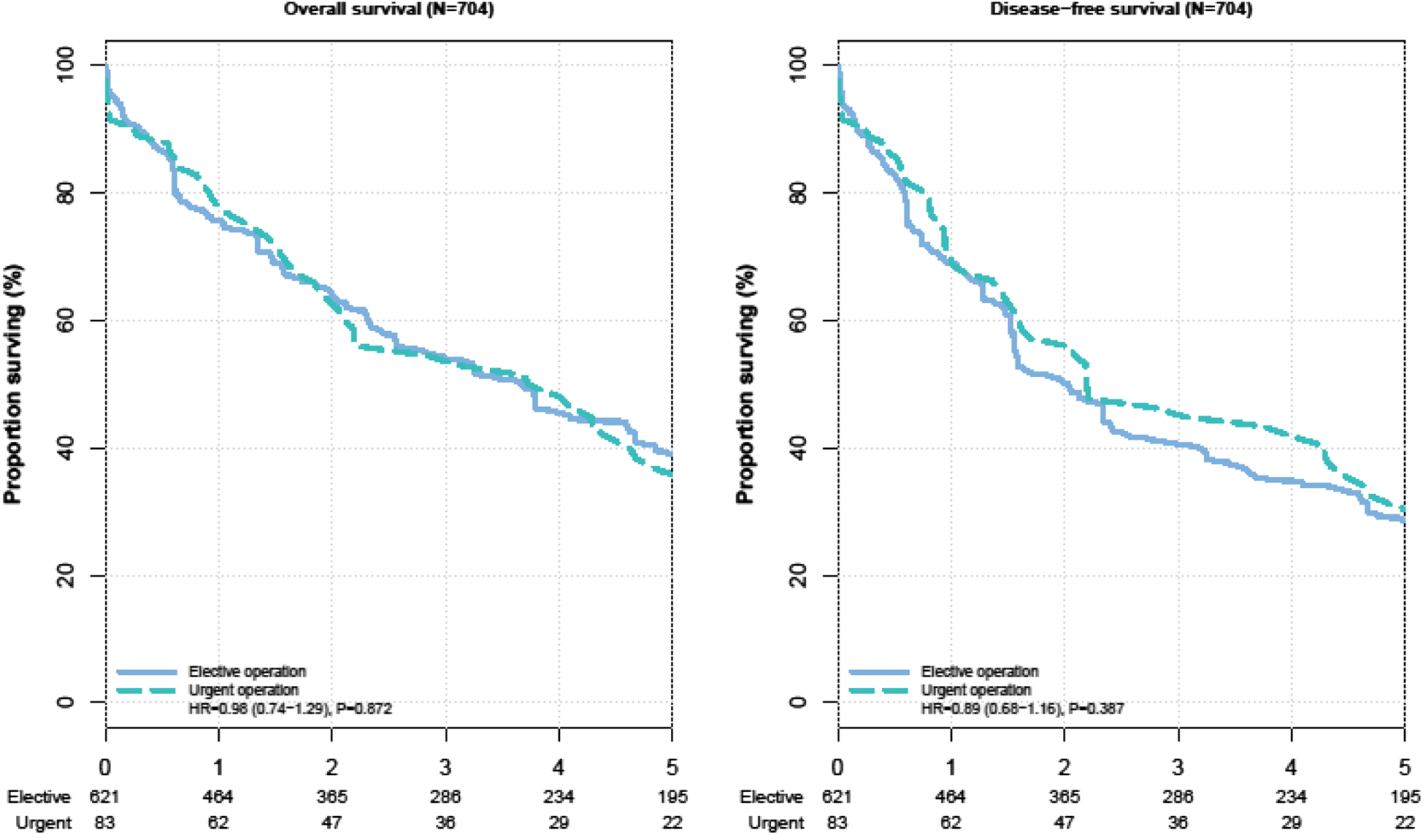
Kaplan–Meier curve for overall and disease-free survival in propensity score adjusted analysis. The number of colorectal cancer patients at risk are given below each plot. Survival curves are provided with 95 % confidence intervals

The propensity score for patients who underwent urgent operation was 0.22 ± 0.16 compared to 0.10 ± 0.09 in patients who underwent elective operation (*P* < 0.001), thus indicating a strong bias regarding the patient characteristics in the two groups. When performing the propensity score matching procedure, 42 patients with elective operation and one patient with urgent operation had to be excluded because their characteristics could not be matched with patients from the other group. Hence, the propensity score-matched analysis was based on 704 patients. After the matching procedure, the propensity score was virtually the same in the two patient groups (0.21 ± 0.15 vs. 0.21 ± 0.15, *P* = 0.969). Fig. 2 displays the change in the distribution of the propensity score due to the matching procedure. After adjusting the data according to the propensity score analysis, urgent versus elective operation did not influence overall survival (HR = 0.98, 95 % CI: 0.74 to 1.29), *P* = 0.872) and disease-free survival (HR = 0.89, 95 % CI: 0.68 to 1.16, *P* = 0.387) (Fig. 3).

## Discussion

The present study is the first study using both Cox regression analyses as well as propensity scoring methods to assess the impact of urgent versus elective operation on overall and disease-free survival in patients undergoing resection for colorectal cancer. This study provides evidence that patient characteristics are strongly biased regarding urgent operation. Optimal adjustment for this bias demonstrates no significant differences in overall and disease-free survival neither after multivariable Cox regression nor after propensity score-adjusted analyses.

In our study, 11 % of patients underwent urgent operation for colorectal cancer. This is comparable to other published investigations [3, 8, 23], although some studies report emergency presentation rates of up to 30 % [1, 2, 6, 10]. However, these studies did not clearly state whether patients were operated within hours or have been operated days after hospital admission. One of the strengths of our study is the clear definition of urgent surgery. This may account for the rather low percentage of patients in this group.

Urgent operation was not associated with poor survival in our study. Although unadjusted risk analysis did show reduced survival following urgent operation, this difference was no longer of statistical relevance after risk-adjustment. The increased risk observed in unadjusted analysis is clearly due to differences in baseline characteristics and not due to the urgent operation itself. Our results are supported by findings from recent studies which showed no statistical differences in long term survival [5, 7, 9, 10]. These reports differ from some larger studies that reported poorer survival for colorectal cancer patients presenting as an emergency [1–3, 6]. But it is not clear from these studies to what extent adjuvant therapy was administered and if so, differences were observed between the investigated groups. Furthermore the information if patients with neoadjuvant therapy were included in the respective studies is not provided. In our study, all patients receiving neoadjuvant treatment were excluded and administration of adjuvant chemotherapy was not different between the two groups. Adjuvant chemotherapy was confirmed as an independent favorable prognostic factor for overall survival as well as the number of harvested lymph nodes. Age, tumor location, resection status, tumor stage, and affected lymph nodes as well as tumor grade were confirmed to be independent prognostic factors for overall and disease free survival (Table 2). Besides these well known prognostic factors, patients receiving urgent surgery significantly more often presented with tumor perforation (Table 1). This is explained by the fact that peritonitis on the basis of perforated colorectal cancer is a common cause of emergency department presentation [24]. However, tumor perforation failed to be a prognostic factor for survival in our analysis. This is most likely based on the fact that not only free intraperitoneal rupture of the tumor was included in this group but also tumors showing localized perforation or those with penetration of the serosal surface in histological analysis.

Surprisingly, lymph node yield was higher in patients undergoing urgent operation in the present study (Table 1). Unfortunately, most of the published studies do not state the amount of resected lymph nodes [1, 2, 4, 8–12]. This is somewhat surprising, giving the fact that the number of harvested lymph nodes is crucial for staging of colorectal cancer patients because lymph node involvement represents the strongest prognostic factor and serves as the most important selection criterion for adjuvant chemotherapy [25]. Additionally, the number of surgically removed and pathologically assessed lymph nodes influences the staging accuracy and impacts overall survival [26, 27]. As a consensus standard, a minimum of 12 examined lymph nodes per patient is therefore recommended for accurate staging. In the present investigation 88.1 % of urgent surgery and 76.5 % of elective surgery patients had ≥ 12 lymph nodes resected (*p* = 0.016). This demonstrates that proper oncologic resection is achievable in urgent operations. Furthermore, the comparable quality of oncologic resection in both groups may be an explanation for the unobserved differences in overall and disease-free survival. It is well known from the literature that both, surgeon as well as hospital specific specialisation and caseload are important predictors for outcome after colorectal cancer resection what seems to apply also for these results [28, 29].

Our study has several limitations. First, this is a retrospective cohort study and not a randomized controlled trial. However, it is not possible to perform a randomized trial for this research question. A cohort study adopting Cox regression analyses as well as propensity-scoring methods probably represents the most appropriate and highest-evidence level study design. Second, while we did comprehensive risk-adjustment for observed confounders, potential bias due to unknown or unobserved confounders, such as American Society of Anaesthesiologist (ASA) grade, comorbidities and adherence to cancer related follow-up care, cannot be completely excluded. And last, all operations in this study were performed or supervised by experienced surgeons of a tertiary care center, what may also have influenced survival rates.

## Conclusion

In summary, urgent colorectal cancer resection does not influence overall and disease-free survival after risk-adjusting in multivariable Cox proportional as well as propensity score analyses. The observed association between urgent operation and oncologic outcome is caused by differences in patient and tumor characteristics. Urgent operation itself is not a risk factor and colorectal cancer resection should therefore not be postponed for oncologic outcome reasons.

## Abbreviations

ASA: American Society of Anaesthesiologist
HR: hazard ratio
